# Increased CD83 expression of CD34-positive monocytes in donors during peripheral blood stem cell mobilization in humans

**DOI:** 10.1038/s41598-019-53020-9

**Published:** 2019-11-11

**Authors:** Hideki Nakasone, Misato Kikuchi, Koji Kawamura, Yu Akahoshi, Miki Sato, Shunto Kawamura, Nozomu Yoshino, Junko Takeshita, Kazuki Yoshimura, Yukiko Misaki, Ayumi Gomyo, Aki Tanihara, Machiko Kusuda, Masaharu Tamaki, Shun-ichi Kimura, Shinichi Kako, Yoshinobu Kanda

**Affiliations:** Division of Hematology, Jichi Medical University Saitama Medical Center, Saitama, Japan

**Keywords:** Immunology, Translational research

## Abstract

CD34-positive monocytes (CD34+mono) have recently been identified in grafts mobilized by granulocyte-colony stimulating factor. We analyzed transplant outcomes of 73 patients whose donor’s peripheral blood cells were cryopreserved during mobilization. CD34+mono was detected more frequently in male donors (67% vs. 40%, P = 0.03), while the detection of CD34+mono in donors was not associated with the patient background. Although there was no significant difference in overall survival in the whole cohort, the detection of CD34+mono in donors were significantly associated with a decreased risk of non-relapse mortality (HR 0.23, P = 0.035). Fatal infectious events tended to be less frequent in donors with CD34+mono. Gene expression profile analyses of CD34+mono in humans revealed that the expressions of pro-inflammatory cytokines like IL6, CCL3, IL8, VEGFA, and IL1A were elevated in CD34+mono, and those cytokines were enriched in the immune response, especially against infectious pathogens in the gene ontology analyses. In addition, the expression of CD83 was specifically increased in CD34+mono. It might play a role of antigen presentation in the immune network, leading in a clinical benefit against infections. Further investigations will be required to confirm the biological functions and clinical roles of CD34+mono in transplantation.

## Introduction

Allogeneic haematopoietic cell transplantation (HCT) has been recognised as a radical strategy for haematological diseases, and its indication has spread partially due to the progress with donor sources such as peripheral blood stem cells (PBSC) mobilized by granulocyte-colony stimulating factor (G-CSF)^1^. Over the past decade, PBSC has been used most widely as a donor source^1^.

A graft of PBSC mobilized by G-CSF is known to contain many more T-cells than bone marrow or cord blood^1^. The 10-fold-greater number of T-cells in a PBSC graft might raise a concern about graft-versus-host disease (GVHD)^1^. In fact, PBSC is known to increase the risk of chronic GVHD compared with other sources^2–6^. However, the adverse impact of PBSC on acute GVHD is still controversial, and the risk is not always increased by PBSC^5,7,8^.

The reason for the lower incidence of acute GVHD than we expected remains unclear, but the emergence of immune-modulating cells during G-CSF-mobilization, called myeloid-derived suppressor cells (MDSCs), has been suggested to explain this phenomenon^9–11^. Recently, CD34-positive monocytes (CD34+mono) have newly been reported in G-CSF-mobilized PBSC grafts, and have been demonstrated to exhibit immunosuppressive activity in animal models^12^. However, it is unclear whether the detection of CD34+mono in donors could be associated with a decreased incidence of GVHD or non-relapse mortality (NRM) after HCT in actual clinical settings. The features of human CD34+mono in terms of gene expression are also unknown. Thus, this study explored the impact of the detection of CD34+mono in donor peripheral blood on clinical outcomes after HCT, and demonstrated the gene expression profiles (GEP) of CD34+mono compared with CD34+ cells and monocytes.

## Results

### Detection of CD34-positive monocytes in donor peripheral blood during mobilization in association with the characteristics of the patient and donor

**Figure 1:**
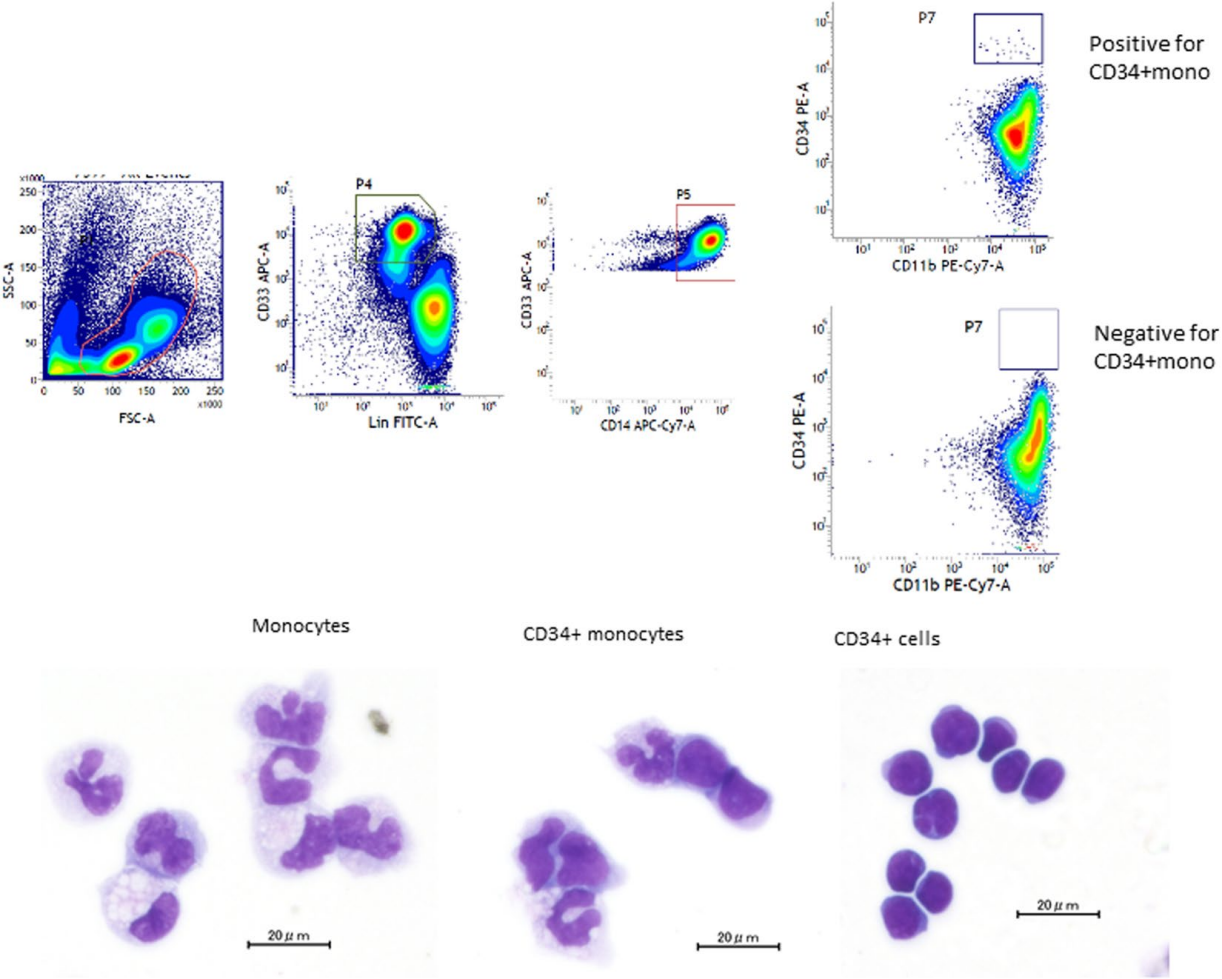
Identification and appearance of CD34-positive monocytes. CD34+mono was defined as Lin^−^CD34^high^CD14^+^CD11b^+^CD33^+^ cells. Wright-Gimsa staining was performed for cyto-centrifuged cells after sorting. Each scale bar denotes 20 µm.

CD34+mono was defined as Lineage(Lin)^−^CD34^high^CD14^+^CD11b^+^CD33^+^ cells (Fig. 1). Of the 73 donors, CD34+mono was detected in 37 (51%), and the median frequency was 0.7% (range: 0.1–2.4%) of all CD34+cells in the donors with CD34+mono. CD34+mono was detected more frequently in male donors (67% vs. 40%, P = 0.03), while there were no differences in the distributions of donor age and body weight (Table 1). The dose of CD34+cells collected at the first day of the stem cell collection tended to be higher in donors who tested positive for CD34+mono, although it was not significant (P = 0.09). Patient diseases included acute leukaemia in 48, lymphoma in 9, myelodysplastic syndrome in 7, and others in 9. Thirty-one patients had a high-risk disease. The detection of CD34+mono in donor peripheral blood was not associated with the patient background, including patient age, patient gender, disease and risk, human leukocyte antigen (HLA)-mismatch, conditioning intensity, or GVHD prophylaxis (Table 1). The median follow-up duration among the survivors was 1087 days.

**Table 1:** Detection of CD34-positve monocytes and characteristics of patients and donors.

Donor background		CD34+ monocytes		P-value
		negative (n = 36)	positive (n = 37)	
Donor Age	Median (range)	41 (17–64)	39 (14–63)	0.78
Donor Gender	Female	26	17	0.03
	Male	10	20	
Body Weight	Median (range)	58.0 kg (40.8–88.3)	62.5 kg (45.4–96.0)	0.51
Dose of CD34+cells collected at the first day	Median (range)	1.64 × 10^8^ cells (0.27–7.78)	2.07 × 10^8^ cells (0.38–11.9)	0.09
Total dose of CD34+cells during their mobilization	Median (range)	2.64 × 10^8^ cells (1.27–7.78)	2.20 × 10^8^ cells (1.40–11.9)	0.45
% of peripheral monocytes at harvest	Median (range)	4.2% (1.4–9%)	4.80% (2.2–13.2%)	0.08
**Patient background**				
Patient Age	Median (age)	44 (20–66)	41 (19–66)	0.96
Patient Gender	Female	13	19	0.24
	Male	23	18	
Disease	ALL	7	9	0.47
	AML	13	19	
	ML	6	3	
	MPN MDS	5	2	
	others	5	4	
Disease risk	standard	22	20	0.64
	high	14	17	
Prior transplant	no	32	28	0.22
	yes	4	9	
CMV sero-positivity	negative	3	5	0.71
	positive	33	32	
HLA-match	match	16	21	0.35
	mismatch	20	16	
GVHD prophylaxis	CsA-based	35	35	1
	Tac-based	1	2	
*In vivo* T cell depletion	no	24	23	0.81
	Yes	12	14	
Conditioning	MAC	27	27	1
	RIC	9	10	

AML, acute myelogeneous leukemia; ALL, acute lymphoblastic leukemia; ML, malignant lymphoma; MDS, myelodysplastic syndrome; MPN, myeloproliferative neoplasm; CMV, cytomegalovirus; GVHD, graft-versus-host disease; CsA, cyclosporine; TAC, tacrolimus; MAC, myeloablative conditioning; RIC, reduced-intensity conditioining.

### Clinical outcomes

#### Overall survival (OS) according to the detection of CD34+mono in donors

**Figure 2:**
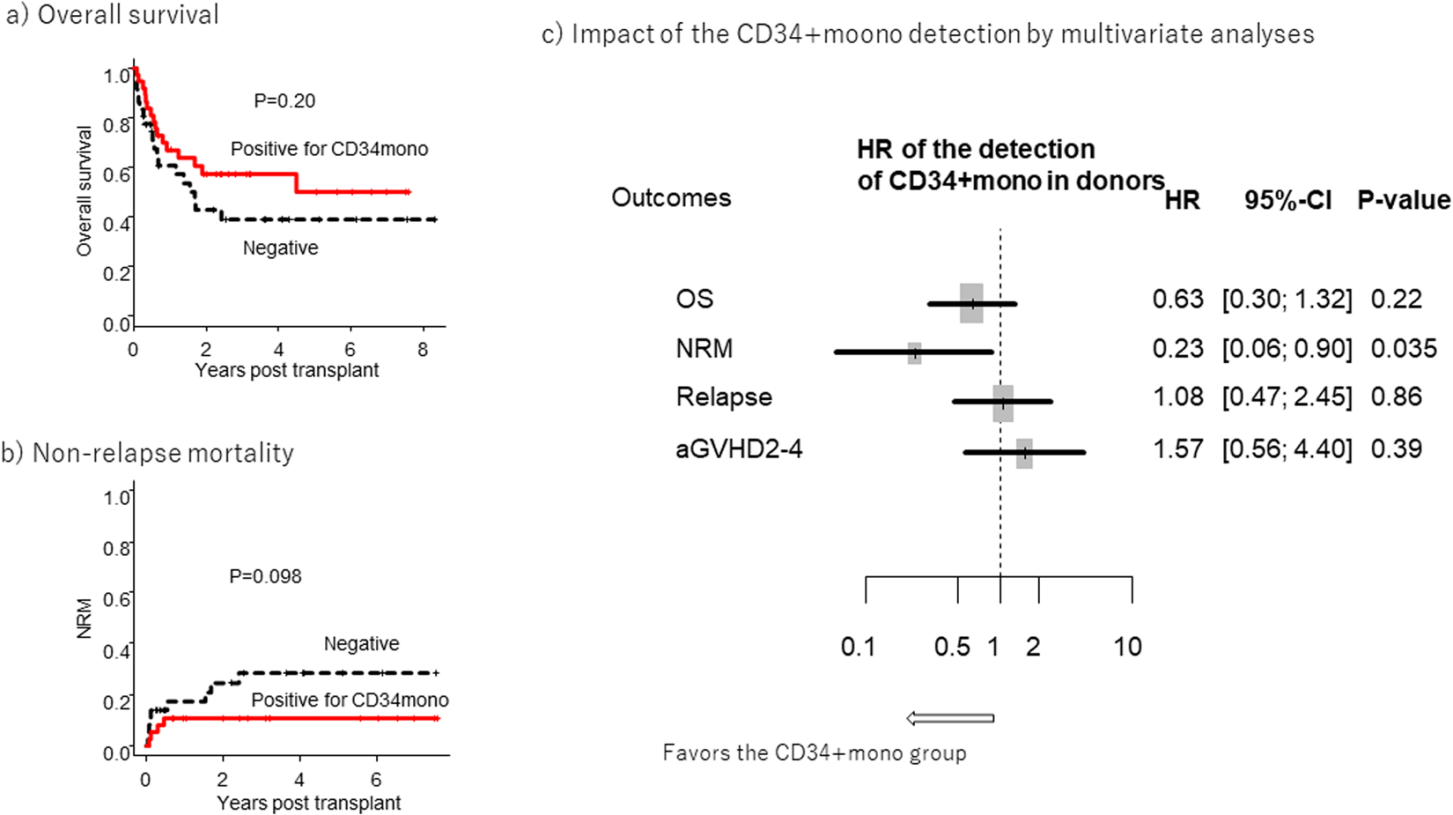
Clinical outcomes according to the detection of CD34+monocytes in the whole cohort: (**a**) overall survival (OS), (**b**) non-relapse mortality (NRM), and (**c**) forest plots for the impact of the detection of CD34+mono on clinical outcomes by multivariate analyses. Multivariate analyses were performed by a Cox proportional hazard model, and the hazard ratio (HR) of the detection of CD34+mono was adjusted for patient and donor age (≥50 years), gender, disease risk, conditioning intensity, and *in vivo* T-cell depletion.

The 3-year OS was 58% (95% confidence interval (CI): 40–72%) in the donor group with CD34+mono vs. 39% (95% CI: 22–56%) in the donor group without CD34+mono (P = 0.20, Fig. 2a). In a multivariate analysis of the whole cohort, the detection of CD34+mono was not associated with superior survival (hazard ratio (HR) 0.63, [95% CI: 0.30–1.32], P = 0.22). We subsequently checked the impact of the CD34+mono detection in sub-cohorts stratified by age, disease risk, conditioning intensity, and T-cell depletion (Fig. S1a–d). The 3-year OS of the CD34+mono group was significantly higher in the sub-cohort with age <50 (69% vs. 40%, P = 0.029, Fig. S1a).

#### Non-relapse mortality (NRM) and relapse

In the whole cohort, the 3-year NRM tended to be lower in the donor group with CD34+mono (11% [95% CI: 3–23%] vs. 29% [95% CI: 14–46%], P = 0.098, Fig. 2b). Multivariate analyses revealed that the detection of CD34+mono was significantly associated with a decreased risk of NRM (HR 0.23 [95% CI: 0.06–0.90], P = 0.035). Especially, the favourable effect of the detection of CD34+mono on NRM seemed significantly apparent in the sub-cohort of age <50 (0% vs. 26%, P = 0.012), standard-risk diseases (5% vs. 33%, P = 0.039), or RIC (10% vs. 75%, P = 0.010) (Fig. S2a–d).

Regarding the incidence of relapse, no differences were observed between donor groups with and without CD34+mono in the whole cohort (50% vs. 44%, P = 0.92, Fig. S3a).

#### Adverse events and cause of death

Next, we explored what kinds of adverse events contributed to the higher NRM in the donor group without CD34+mono. There were no differences in the incidence of grade II-IV acute GVHD (32% [95% CI: 18–48%] vs. 19% [95% CI: 8–34%], P = 0.24, Fig. S3b) or in the neutrophil recovery of >0.5 × 10^9^/L (89% [95% CI: 72–96%] vs. 92% [95% CI: 74–98%] at 30 days after HCT, P = 0.89, Fig. S3c) between the groups with and without CD34+mono. The impact of the detection of CD34+mono on clinical outcomes were summarized in Fig. 2c. The differences in clinical parameters for immune recoveries were further checked, but no significant differences were observed in the levels of CD4+Tcells, CD8+Tcells, and IgG between the groups, although the median values seemed higher in the CD34+mono group (Fig. S4).

The distribution of cause of death was significantly different between the donor groups with and without CD34+mono (P = 0.031, Table S1), and infectious deaths were infrequent in donors with CD34+mono (6% vs. 37%).

### Gene expression profile (GEP) of CD34-positive monocytes compared with other CD34-positive cells and monocytes

#### Hierarchical clustering map

**Figure 3:**
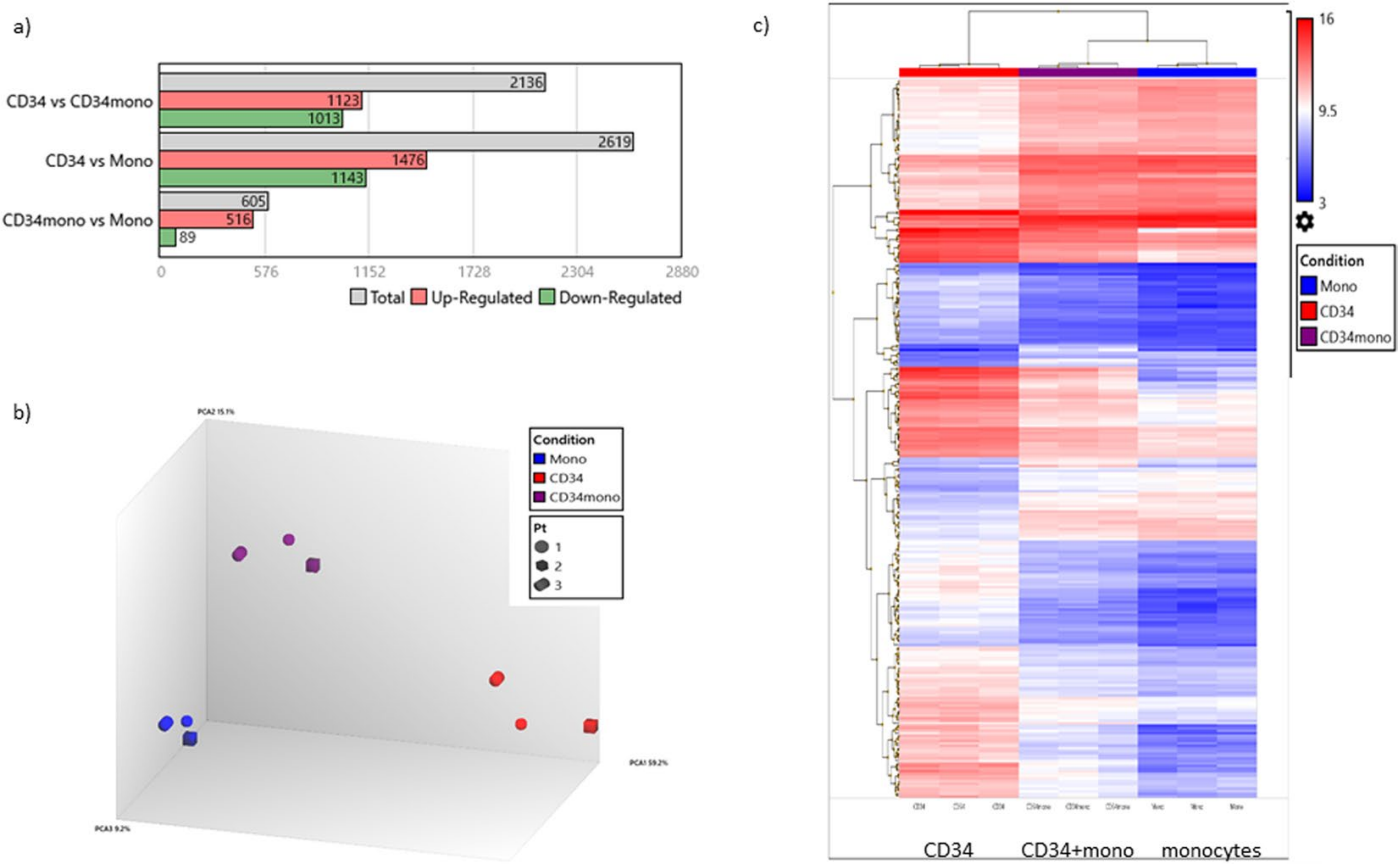
Gene expression profile analyses for CD34+mono, CD34+cells, and monocytes (n = 3 in each). (**a**) Number of differentially expressed genes among CD34+mono, CD34+cells, and monocytes. (**b**) Principle component analysis mapping. (**c**) Hierarchical clustering for the gene expression of CD34+mono, CD34+cells, and monocytes filtered by a conditional FDR F-test < 0.0005.

Based on the clinical observation described above, we hypothesized that CD34+mono might have an additional potential against infections compared with monocytes and CD34+cells, and thus performed GEP analyses. CD34+mono generally resembled monocytes in appearance (Fig. 1). Actually, the number of differentially expressed genes was smaller in the pair of CD34+mono vs. monocytes compared with the other pairs (Fig. 3a), suggesting that the features of CD34+mono might be close to those of monocytes. However, a principle component analysis suggested that CD34+mono was grouped as an independent entity (Fig. 3b). In addition, many genes of CD34+mono seemed to be expressed intermediate between CD34+cells and monocytes (Fig. 3c).

#### Gene ontology (GO) and pathway analyses

**Figure 4:**
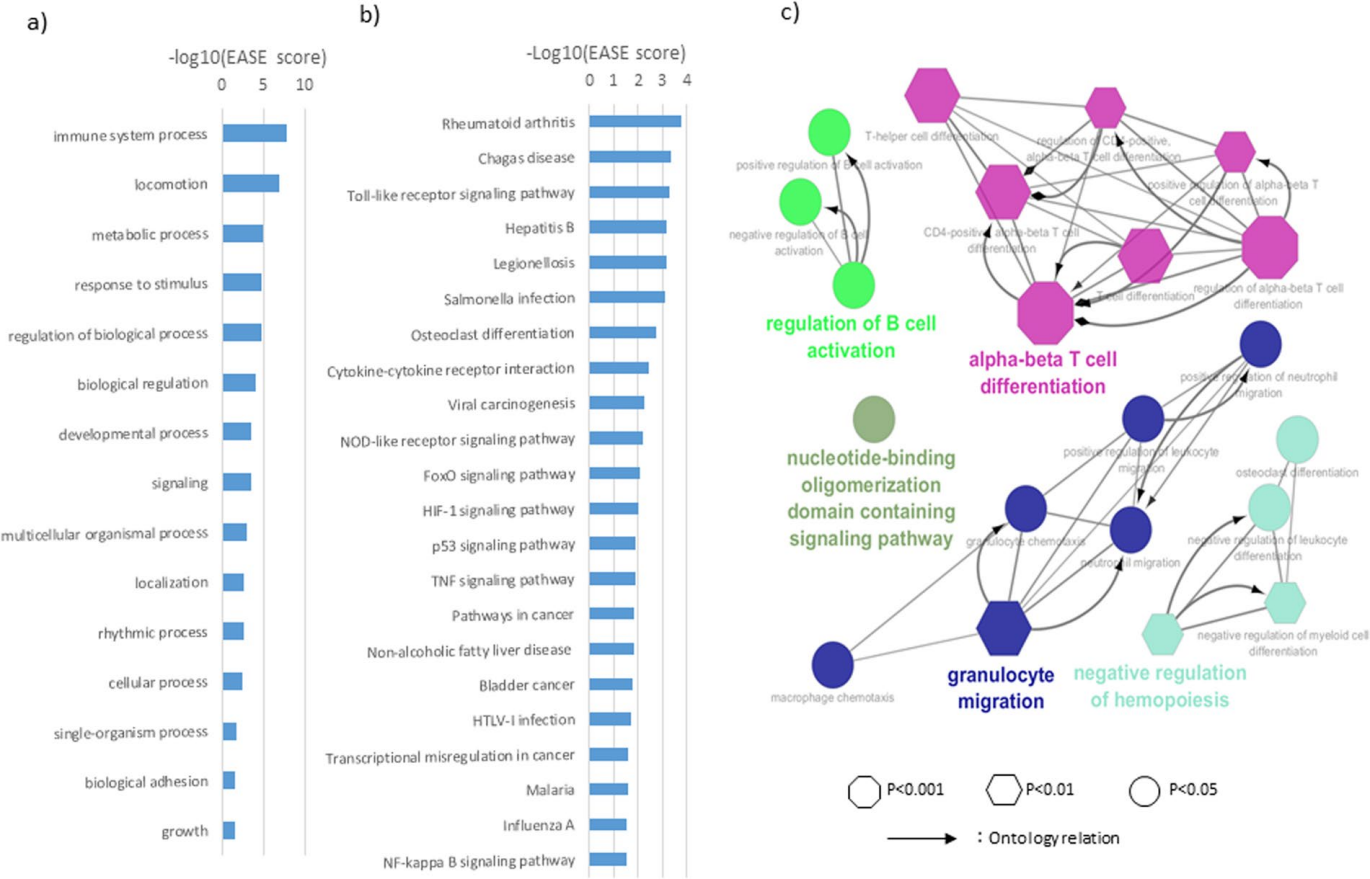
Gene ontology (GO) and pathway analyses for CD34+mono-specific genes. (**a**) GO biological process 1, (**b**) KEGG pathways, (**c**) GO focusing on immune system processes and their relationship.

The current GO analyses included the 203 genes that were differentially expressed in CD34+mono, filtered by F-test values of <0.05 and 2 or more-fold changes compared with CD34+cells and monocytes (Data File S1). The differentially expressed genes were most frequently involved in the biological process “immune system process”, followed by “locomotion”, “metabolic process” and “response to stimulus” (Fig. 4a, Data File S2). Furthermore, the pro-inflammatory genes significantly increased in CD34+mono, like IL6, CCL3, IL8, VEGFA, and IL1A, were mapped in immunological pathways like toll-like receptor signalling as well as in response to pathogens (Fig. 4b, Data File S3). Next, when we focused on the GO of the immune system process, the differentially expressed genes in CD34+mono were significantly involved in T-cell differentiation, followed by granulocyte migration and regulation of haematopoiesis (Fig. 4c). In summary, the genes differentially expressed in CD34+mono were enriched in the immune response to infections, and were associated with T-cell differentiation process.

#### Protein-protein interactions (PPI) network

**Figure 5:**
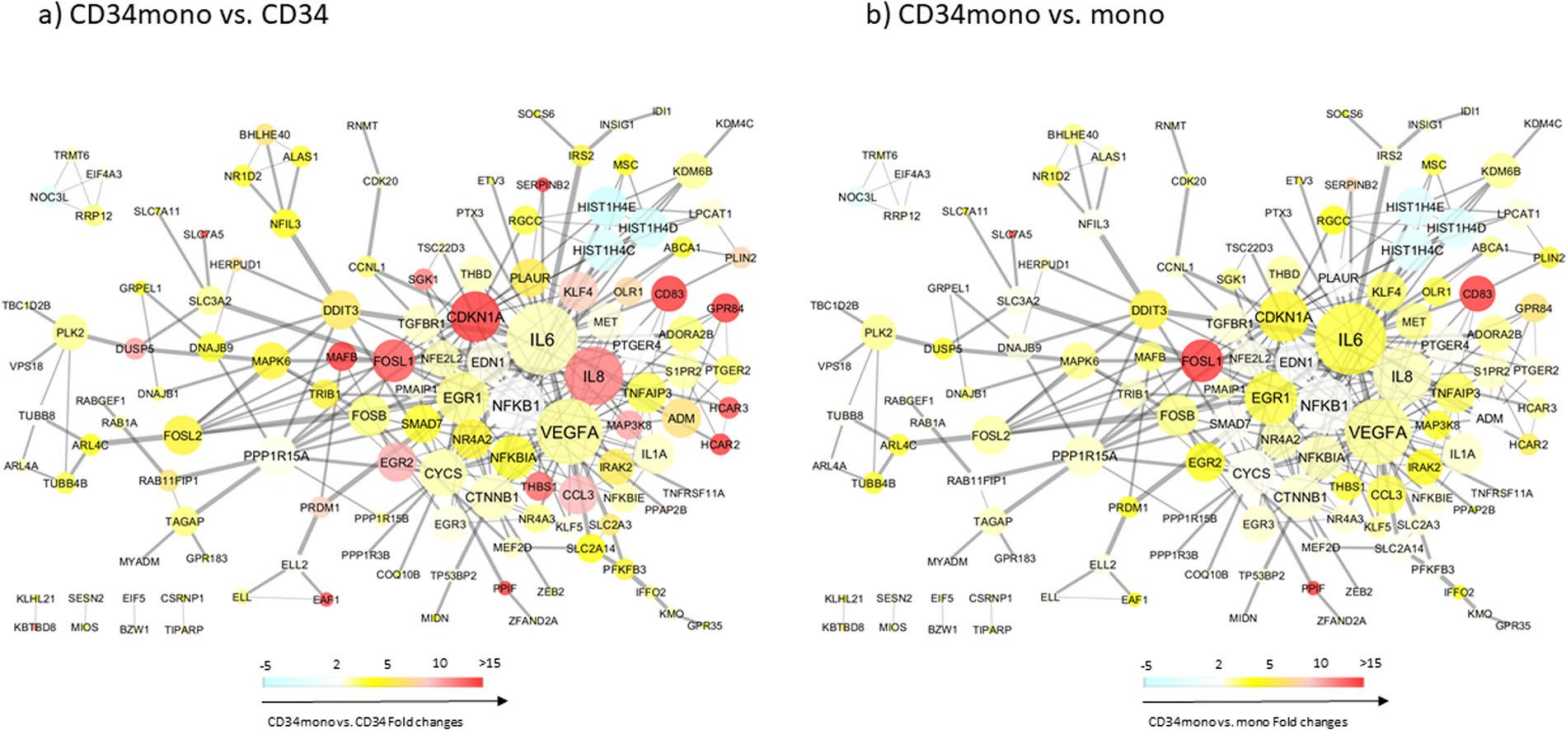
Protein-protein interactions network and gene expression fold changes, colored according to the fold changes of (**a**) CD34-positive monocytes vs. CD34 cells, or (**b**) CD34-positive monocytes vs. monocytes.

PPI network analyses of the 203 differentially expressed genes in CD34+mono demonstrated that 128 had close connections with others. According to degree centralities of ≥15, hub genes of the CD34+mono network were IL6, VEGFA, IL8, NFkB1, EGR1, CDKN1A (p21), and CYCS (the top 30 genes according to other criteria are shown in Table S2). IL6, IL8, and VEGFA are pro-inflammatory cytokines. NFkB1 and EGR1 are transcriptional factors, while CDKN1A and CYCS are regulators of apoptosis or cell cycle. In addition, while simultaneously focusing on the fold changes between CD34+mono vs. CD34+cells (Fig. 5a) or vs. monocytes (Fig. 5b), we found that CD83 (a membrane protein and immunoglobulin superfamily that regulates antigen presentation) and FOSL1 (a kind of regulator of cell proliferation, differentiation, and transformation) were specifically elevated in CD34+mono with a relatively higher degree of centrality, suggesting that these genes would work centrally and specifically in the PPI network of CD34+mono. On the other hand, PPIF (a regulator of apoptotic or necrotic cell death) and SLC7A5 (a light unit of heterodimeric amino acid transporters) might be consequently increased, because of their peripheral connections in the PPI network of CD34+mono.

### Antigen-presenting potential: Response of CD3+T-cells to cytomegalovirus peptides in the presence or absence of CD34+mono

**Figure 6:**
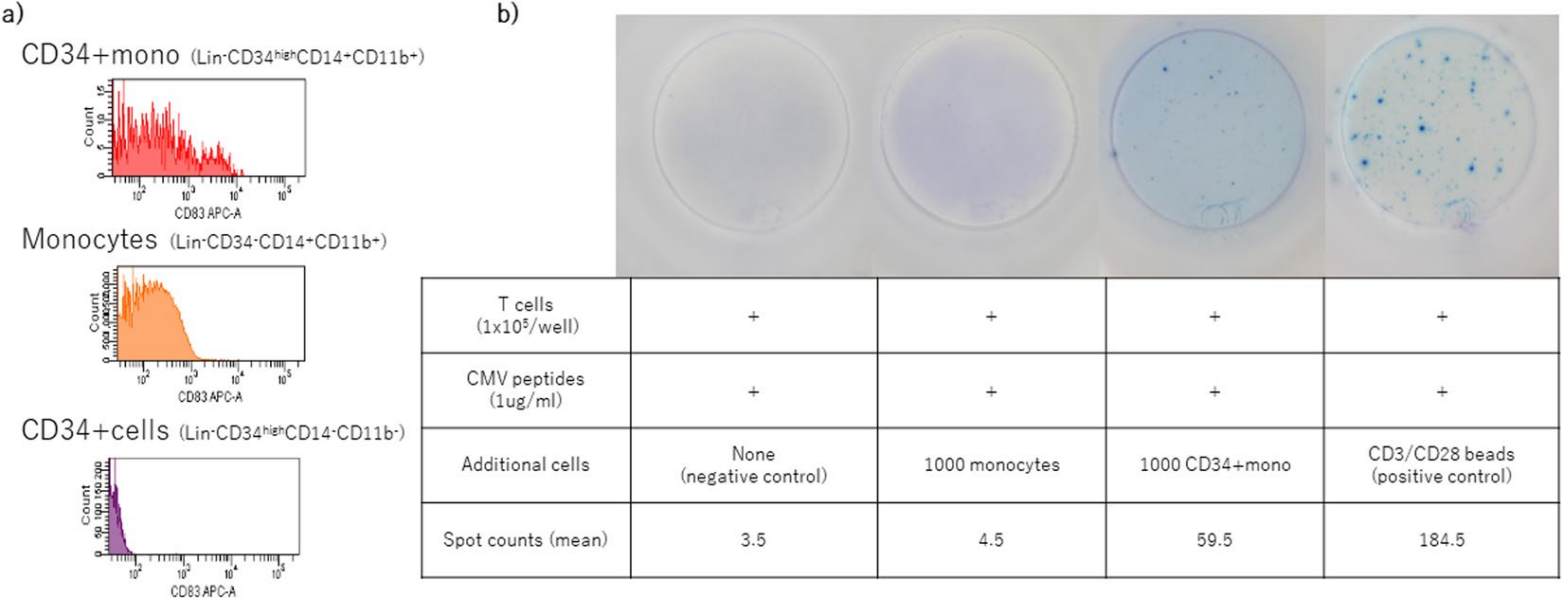
Surface expression of CD83 and the potential of antigen presentation of CD34+mono. (**a**) Surface expression of CD83 in CD34+mono, monocytes, and CD34+cells from a graft with CD34+mono as a representative. (**b**) ELISpot assay for the secretion of interferon-γ by CD3+T cells with or without the presence of CD34+mono in duplicate.

Based on the current GEP, we focused on the increased CD83 expression in CD34+mono. CD83 is known to be specifically expressed in antigen-presenting cells such as dendritic cells (DCs). FACS analyses suggested that not only gene expression but also surface expression of CD83 seemed to be increased in CD34+mono compared with the others (Fig. 6a). Therefore, we hypothesized that CD34+mono might contribute to the response of T-cells through antigen presentation. Enzyme-Linked ImmunoSpot (ELISpot) assays revealed that co-culture with CD34+mono induced interferon-γ secretion from CD3+T-cells, while that with monocytes did not (Fig. 6b).

## Discussion

We assessed the clinical impact of the detection of CD34+mono in donor peripheral blood during G-CSF-mobilization. No significant difference in OS was observed between donor groups with and without CD34+mono in the whole cohort. However, the detection of CD34+mono was significantly associated with a reduced risk of NRM due to the low incidence of fatal infections. Through GEP, we found that the differentially expressed genes in CD34+mono were involved mainly in the pathways of immune processes against pathogens. Additionally, the expression of CD83 was specifically increased in CD34+mono compared with CD34+ cells and monocytes, suggesting that it plays a role in antigen presentation.

CD34+mono was identified as a subtype of monocytic MDSCs by the French group, and was reported to have a potential to induce apoptosis of allogeneic T-cells in mice, leading to immune tolerance through inducible nitric oxide synthase (iNOS)^12^. They found that mice receiving CD34+mono were less likely to develop GVHD in an allogeneic transplant model, and concluded that CD34+mono-derived NO would regulate the allogeneic response by T-cell apoptosis with a subsequent induction of regulatory T-cells (Treg)^12^. Importantly, it should be noted that this inhibitory effect by CD34+mono initially requires T-cell activation by interferon-γ^12^. Although they also observed a low incidence of acute GVHD in HCT from a graft with a higher dose of CD34+mono in humans, the study population was too small (n = 19) to conclude that it offered any clinical advantage. In the current larger cohort (n = 73), we did not confirm a favourable impact on acute GVHD. This may have been due to their heterogeneous backgrounds or the fact that we could not assess the total dose of CD34+mono in graft.

On the other hand, we alternatively observed a reduced NRM due to the low incidence of fatal infections in the donor group with CD34+mono. Actually, GEP analyses revealed that pro-inflammatory cytokines like IL1, IL6, IL8, and VEGF were significantly increased in CD34+mono compared with monocytes and CD34+cells, which were considered to be involved in the pathways of innate and adaptive immunity against pathogens. These GEP and GO analyses help us to speculate on the reasons for the favourable benefit in the CD34+mono group.

To the best of our knowledge, this is the first study to demonstrate the possibility that CD34+mono plays a role in antigen presentation as well as the GEP of CD34+mono in humans. We found that the expression of CD83 was increased in CD34+mono and would act centrally in the PPI network. In general, CD83 is known to be a highly specific marker for mature DCs^13–18^. At least eight subsets of DCs have been identified so far, but their common role in immunity is considered to induce both T-cell-mediated immune response and tolerance^19,20^. Importantly, defence against viral infections requires efficient antigen presentation, and DCs critically sense infections and tissue damage, prime and activate the sequential T-cell response, and eventually polarize T-cell effector cells^19^. In fact, our experiments demonstrated that co-culture with CD34+mono induced interferon-γ secretion from CD3+ T-cells, supporting the potential of antigen presentation by CD34+mono. An additional infusion of CD34+mono after HCT from G-CSF-mobilized donors may become a candidate of cell therapy to prompt immune reconstitution against repeated or refractory infections.

Furthermore, it is also well established that central and peripheral tolerances also require antigen presentation by DCs to CD4+ T-cells to recognize auto-antigens for the generation of thymus-derived or peripherally-induced Tregs^17,19^. Thus, the idea that CD34+mono may be a subtype of DCs or progenitor cells could well explain both observations of the regulatory effect on the allogeneic response following T-cell activation in the previous animal study from France^12^ and the protective potential against fatal infections in the current study.

DCs are considered to originate from haematopoietic cells, and can be generated *ex vivo* from peripheral monocytes with granulocyte-macrophage-CSF, IL4, IL2, and TNF-α^21^. Therefore, monocyte-derived DCs might be a further matured and differentiated cell type of monocytes, instead of differentiating into macrophages. On the other hand, DCs or DC precursors are also believed to be directly generated *ex vivo* from CD34+ progenitor cells^22–24^. Thus, G-CSF-mobilized CD34+mono in humans might be a step in the direct differentiation of CD34+ cells into DCs *in vivo*. Future basic studies will be needed to clarify the difference in functions between CD34+mono and monocyte-derived DCs.

This study had several limitations in addition to the retrospective nature and small cohort. First, we could not assess the absolute counts of CD34+mono in grafts actually infused to patients, as mentioned above. Grafts with a higher dose of CD34+mono may affect NRM as well as GVHD, relapse, and survival. Therefore, we now have a plan to prospectively assess the impact of the cell dose of CD34+mono in actually infused grafts on clinical outcomes in a larger multi-centre cohort. In addition, the cut-off value of CD34+mono detection remains to be elucidated. Second, the patient backgrounds were heterogeneous, and this might have affected the strategy to prevent GVHD and relapse. Third, CD34+mono was a rare population among G-CSF-mobilized peripheral blood. Therefore, the gating threshold for sorting cells may influence the GEP results due to accidental contamination, since our 6-cololr sorting system may not completely delete the spill over of the other cell types and post-sorting re-analyses could not be performed due to the concern on cell count loss. However, we observed similar GEP from different donors, which could support our clinical observation of a protective potential against infections based on bioinformatics.

In conclusion, the detection of CD34+mono in donors during G-CSF mobilization might be associated with a reduced risk of NRM due to a low incidence of fatal infections. The genes differentially expressed in CD34+mono were enriched in the immune response, especially against infections. Furthermore, the increased CD83 in CD34+mono indicated that it might play a role in antigen presentation in the immune response. Since the study cohort was too small to conclude anything, further investigations and validations by large cohorts are required to confirm the biological functions of CD34+mono and its clinical roles after HCT.

## Patients and Methods

### Patient selection and category definitions

Among patients who received HCT from related donors between 2009 and 2016 at our institution, we identified 73 donors whose peripheral blood cells during G-CSF-mobilization were available for flow cytometry analyses. Their PBSCs were mobilized using 400 μg/m^2^/day of filgrastim for 4 to 6 days. PBSC collection was started beginning 4 days after the administration of filgrastim, and was continued until either a targeted dose of PBSC (>2 × 10^6^ CD34+cells/kg) was collected or 6 days after administration.

Conditioning regimens that included total body irradiation (TBI) > 8 Gy, melphalan (Mel) > 140 mg/m^2^, or intravenous busulfan (Bu) Bu ≥ 7·2 mg/kg were classified as myeloablative conditioning (MAC), whereas other regimens were classified as reduced-intensity conditioning (RIC)^25–27^. An HLA-match between a related donor-recipient pair was defined as a 6/6 serological match of HLA-A, -B, and -DR. Standard-risk diseases included chronic myeloid leukaemia (CML) in chronic phase, acute myelogenous leukaemia (AML) or acute lymphoblastic leukaemia (ALL) in complete remission, myelodysplastic syndrome (MDS) other than refractory anaemia with excess blasts, myeloproliferative neoplasms, lymphoma with complete or partial response, multiple myeloma (MM) with complete or very good partial response (VGPR), and aplastic anaemia. High-risk diseases were defined as AML on ALL in non-remission, CML in advanced or blast phase, MDS with excess blasts, lymphoma without any response, MM with <VGPR, and other tumours. This study was conducted under the approval by the institutional review board of Jichi Medical University and all patients and donors in the current study gave their written informed consent for the cryopreservation and analysis of the blood samples in accordance with the Helsinki Declaration.

### Statistical analyses for clinical outcomes

Numerical and categorical variables were compared using the Mann-Whitney U test and Fisher’s exact test, respectively. OS from HCT was calculated by the Kaplan-Meier method with a 95% confidence interval, and compared by the log-rank test. The cumulative incidence of grade II-IV acute GVHD was estimated and compared by Gray’s method, where death or relapse without the event was thought as a competing risk. Relapse and NRM were also estimated by Gray’s method, considering each other as a competing risk. In multivariate analyses, a Cox proportional hazard model was performed. The hazard ratio of the detection of CD34+mono was adjusted for the following clinical variables: patient and donor age (≥50 years), gender, disease risk, conditioning intensity, and *in vivo* T-cell depletion. When a two-tailed P-value of less than 0.05 were obtained, we considered that there was statistical significance. All clinical data manipulations and statistical approaches were conducted with Stata version 12·0 (Stata Corp., College Station, TX) and EZR ver1.36 (Jichi Medical University at http://www.jichi.ac.jp/saitama-sct/SaitamaHP.files/statmedEN.html)^28^. A clinical dataset for survival analyses was provided in Data File S4.

### Isolation of donor peripheral blood cells and staining for CD34-positve monocytes for flow cytometry analyses

Peripheral blood samples were obtained from donors at a median of 4 days after G-CSF administration (range: 3–6). Mononuclear cells were separated by density gradient sedimentation using Lymphoprep™ (Axis-Shield PoC AS), and cryopreserved at −80 °C until use. Cells were incubated with anti-human Lin (Lineage: CD3, CD19, CD56)-FITC, CD34-PE, CD33-APC or CD83-APC, CD11b-PECy7, CD14-APCCy7 monoclonal antibodies, and 7AAD (BioLegend). Data were analysed with FACSverse and FACSuite (BD Biosciences).

CD34+mono was defined as Lin^−^CD34^high^CD14^+^CD11b^+^CD33^+^ cells (Fig. 1). When ≥0.1% of CD34+mono among CD34-positve cells was detected with ≥5 events, the donor was considered to be positive for CD34+mono.

### Sorting of CD34-positive monocytes and extraction of total RNA

From cryopreserved PBSC grafts of three donors, approximately 1 × 10^4^ CD34+mono cells, 3 × 10^5^ CD34-positive cells (CD34+cells, Lin^−^CD34^high^CD33^−^CD14^−^CD11b^−^ cells), and 3 × 10^5^ monocytes (Lin^−^CD34^−^CD33^+^CD14^+^CD11b^+^ cells) were directly sorted into tubes using FACSAria™ II (BD Biosciences). Doublet cells were excluded by plotting forward scatter (FSC) height vs. FSC area as well as side scatter (SSC) height vs. SSC area. After the collection of individual cell types, total RNA extraction was immediately performed with an RNeasy^®^Plus Micro kit (QIAGEN) according to the manufacturer’s instructions. Thereafter, electrophoresis analyses were performed to examine the quality of the extracted RNAs using an Agilent 2100 Bioanalyzer (Agilent Technologies) with RNA Pico Chips (Agilent Technologies). If an RNA integrity number (RIN) of >6.5 was achieved, the extracted RNAs were transferred to Takara Bio, Inc. (Otsu, Japan) for microarray analyses.

### Amplification of the extracted RNAs and microarray analyses for gene expression profile

Using 6 ng of the extracted RNA in each sample with a GeneChip™ 3′ IVT Pico Kit (Thermo Fisher Scientific), cDNA was made by reverse transcription, and cRNA was then synthesized and amplified by *in vitro* transcription. After the quality assessments, the cRNA was converted to biotinylated double-stranded cDNA hybridization targets. The product of cDNA was then hybridized into a human Clariom™ S Assay (Thermo Fisher Scientific) with a GeneChip™ Hybridization, Wash, and Stain Kit, and a GeneChip™ Hybridization Oven 645 (Thermo Fisher Scientific) via incubation at 45 °C with rotation at 60 rpm for 16 hours. The arrays were automatically stained and read using a GeneChip™ Fluidics Station 450 and a GeneChip™ Scanner 3000 7G (Thermo Fisher Scientific), according to the protocol provided by the manufacturer. The raw data were analysed based on the algorithm of a Transcriptome Analysis Console (Thermo Fisher Scientific). A conditional false discovery rate (FDR) F-test was used to filter genes, and the threshold was <0.0005 for hierarchical clustering without considering fold changes.

### GO and pathway analyses and PPI network construction

The biostatistical approach was performed according to a previous report^29^. Briefly, enrichment analyses were performed for GO and pathways by KEGG (the Kyoto Encyclopedia of Genes and Genomes) through DAVID (the Database for Annotation, Visualization and Integrated Discovery, http://david.abcc.ncifcrf.gov/)^29^. The differentially expressed genes with F-test values of <0.05 and 2 or more-fold changes in CD34+mono compared with CD34+cells and monocytes were selected for enrichment analyses. The values of –log10 (EASE score, which is a modified Fisher Exact P-value) were used to assess the significance of individual ontology terms or pathways^29^. Furthermore, ClueGO, which is a Cytoscape plug-in App^30,31^, was used to visualize biological terms of the immune process in the functionally grouped network of CD34+mono, and the visualized network was modified by Cytoscape (http://www.cytoscape.org/). Next, the protein-protein interaction (PPI) network in CD34+mono was constructed through STRING (Search Tool for the Retrieval of Interacting Genes/Proteins, http://string-db.org/), which is a biological database^29^. The components of the PPI network were selected with a confidence score of >0.4. The visualized PPI network was also modified by Cytoscape^29^.

### ELISpot assay for the detection of interferon-γ secreted by T-cells in response to cytomegalovirus peptides with or without CD34+mono

Using a G-CSF-mobilized PBSC graft from a donor who was seropositive for cytomegalovirus (CMV), we performed the human interferon-γ ELISpot assay according to the manufacturer’s instructions (MABTECH). Briefly, 15 µg/ml antibodies to detect interferon-γ were coated on the MultiScreen™-IP sterile plate (Millipore) on day1, and the plate was incubated overnight at 4–8 °C. On day2, CD34+mono, monocytes, and CD3+ T-cells were sorted into three tubes, and 100,000 CD3+ T-cells were stimulated with 1 µg/ml of CMV peptide pool (Miltenyi Biotec) with 1,000 cells of CD34+mono or monocytes in duplicate. On day5, 1 µg/ml of the biotin-conjugated detection antibodies and diluted streptavidin-HRP in 1:500 were applied, and tetramethylbenzidine (TMB) substrates (MABTECH) were used to develop the spots. An ELIPHOTO counter was used to count the spots (MINERVA TECH).

## Supplementary information

Sup documents

Dataset S1-4

## Data Availability

The data discussed in this publication have been deposited in NCBI’s Gene Expression Omnibus and are accessible through GEO Series accession number GSE126160 (https://www.ncbi.nlm.nih.gov/geo/query/acc.cgi?acc=GSE126160).
