# Supplementary material for: Increased CD83 expression of CD34-positive monocytes in donors during peripheral blood stem cell mobilization in humans
Source: Sci Rep. 2019 Nov 11;9:16499. doi: 10.1038/s41598-019-53020-9 (PMC6848192; doi:10.1038/s41598-019-53020-9)
Supplement: Supplementary file 1 — Sup documents [file 41598_2019_53020_MOESM1_ESM.pdf]

Supplementary Information for

Increased CD83 expression of CD34-positive monocytes in donors during peripheral blood stem cell mobilization in humans

Hideki Nakasone[1†], Misato Kikuchi[1†], Koji Kawamura[1], Yu Akahoshi[1], Miki Sato[1], Shunto Kawamura[1], Nozomu Yoshino[1], Junko Takeshita[1], Kazuki Yoshimura[1], Yukiko Misaki[1], Ayumi Gomyo[1], Aki Tanihara[1], Machiko Kusuda[1], Masaharu Tamaki[1], Shun-ichi Kimura[1], Shinichi Kako[1], and Yoshinobu Kanda[1*]

[†]These authors contributed equally.

*Correspondence

[1] Division of Hematology, Jichi Medical University Saitama Medical Center, Saitama, Japan

Correspondence: Yoshinobu Kanda, MD/PhD

Division of Hematology

Jichi Medical University Saitama Medical Center,

1-847 Amanuma-cho Omiya-ku, Saitama, 330-8503, Japan

E-mail: ycanda-tky@umin.ac.jp

Tel: +81-48-647-2111

This PDF file includes:

Tables. S1 to S2
Figs. S1 to S4
Captions for data files S1 to S4

Other supplementary materials for this manuscript include the following:

Data files S1 to S4

Table S1. Causes of death

Cause of death	Negative for CD34mono n (%)	Positive for CD34mono n (%)	P-value
Relapse	7 (37%)	11 (69%)	0.031
Infections	7 (37%)	1 (6%)	
Lung complications	2 (10%)	4 (25%)	
Others	3 (16%)	0 (0%)	

*Others include hemorrhage, thrombocytic microangiopathy, and hepatic failure.
* P-value was calculated by Fisher's exact test.

Table S2. Important genes through PPI network

	Gene	Degree	Gene	Betweenness centralities	Gene	Subgraph centralities
Top1	IL6	37	IL6	4491.458	IL6	37303.59
Top2	VEGFA	32	VEGFA	2436.612	VEGFA	30177.77
Top3	IL8	25	PPP1R15A	1576.261	IL8	25469.17
Top4	NFKB1	23	DDIT3	1520.716	NFKB1	22105.16
Top5	EGR1	20	CYCS	1485.429	EGR1	17109.54
Top6	CDKN1A	18	FOSL1	1431.147	CDKN1A	13894.45
Top7	CYCS	15	CDKN1A	1255.944	CYCS	9865.219
Top8	CTNNB1	14	EGR1	1228.879	NFKBIA	9834.781
Top9	NFKBIA	12	FOSL2	1095.455	CTNNB1	9443.419
Top10	FOSL1	11	NFKB1	1032.743	EDN1	8111.304
Top11	EDN1	11	IL8	944.8427	IL1A	6301.272
Top12	FOSB	10	FOSB	883.645	CCL3	5438.84
Top13	HIST1H4C	10	CTNNB1	841.8234	FOSL1	4580.178
Top14	PPP1R15 A	10	MAPK6	796.7733	TNFAIP3	4479.766
Top15	HIST1H4D	9	PRDM1	700.1192	MET	4395.444
Top16	HIST1H4E	9	ARL4C	689.3618	SMAD7	4055.011
Top17	IL1A	9	IRS2	676	PTGER4	3959.053
Top18	TNFAIP3	9	NFIL3	672	ADM	3594.76
Top19	DDIT3	8	PFKFB3	672	PLAUR	3419.257
Top20	PTGER4	8	SLC3A2	656.691	THBD	2982.011
Top21	CCL3	8	DUSP5	617.8048	HIST1H4C	2951.297
Top22	FOSL2	8	SLC2A14	502.5616	NFE2L2	2923.291
Top23	ADM	8	PLK2	488	KLF4	2709.166
Top24	KLF4	8	TAGAP	454	TGFBR1	2549.535
Top25	NR4A2	8	CCNL1	452	HIST1H4D	2549.239
Top26	EGR2	8	RAB11FIP 1	452	HIST1H4E	2549.239
Top27	SMAD7	7	ELL2	452	IRAK2	2209.376
Top28	PLAUR	7	IFFO2	452	FOSB	2208.41
Top29	TGFBR1	7	SLC2A3	390.114	THBS1	2062.288
Top30	MET	6	HIST1H4C	290.4947	S1PR2	2013.595

Supplementary Figure Legends:

Fig.S1. Overall survival according to the detection of CD34+monocytes in donors in the sub-cohorts stratified by a) patient age, b) disease risk, c) conditioning intensity, and d) *in vivo* T cell depletion.

Fig.S2. Non-relapse mortality according to the detection of CD34+monocytes in donors in the sub-cohorts stratified by a) patient age, b) disease risk, c) conditioning intensity, and d) *in vivo* T cell depletion.

Fig.S3. Cumulative incidences of a) relapse, b) grade II to IV acute GVHD, and c) neutrophil recovery according to the detection of CD34+mono in the whole cohort.

Fig.S4. Recoveries of T cells and IgG according to the detection of CD34+mono in donors.
(a) Proportion of CD3+CD4+ T cells at 1, 2, and 3 months after transplantation.
(b) Proportion of CD3+CD8+ T cells at 1, 2, and 3 months after transplantation.
(c) Levels of IgG at at 1, 2, and 3 months after transplantation. The Mann-Whitney test was used for the comparisons.

Figure S1

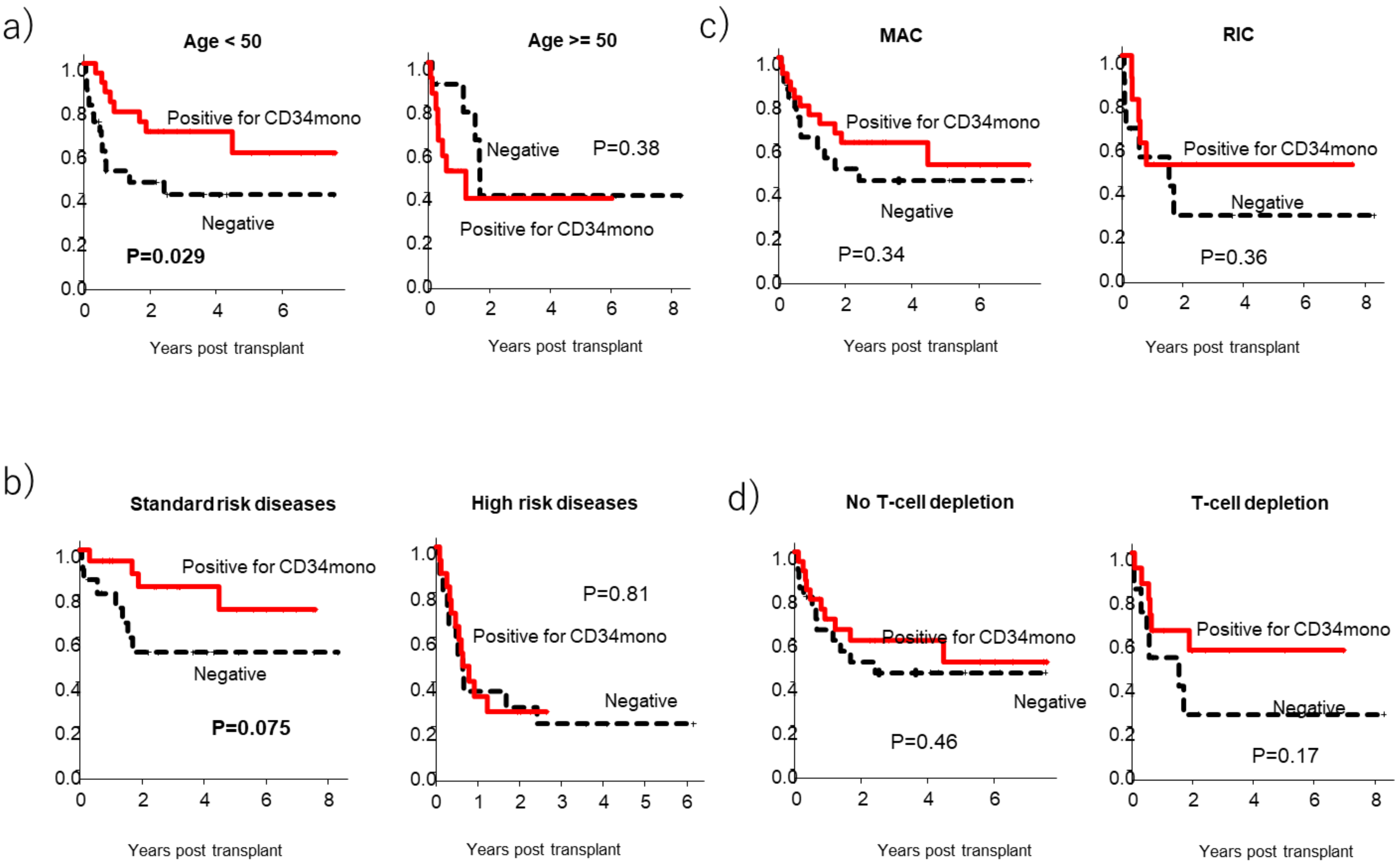
a)
Age < 50
Positive for CD34mono
Negative
P=0.029
Years post transplant
Age >= 50
Negative
P=0.38
Positive for CD34mono
Years post transplant
c)
MAC
Positive for CD34mono
Negative
P=0.34
Years post transplant
RIC
Positive for CD34mono
Negative
P=0.36
Years post transplant
b)
Standard risk diseases
Positive for CD34mono
Negative
P=0.075
Years post transplant
High risk diseases
P=0.81
Positive for CD34mono
Negative
Years post transplant
d)
No T-cell depletion
Positive for CD34mono
Negative
P=0.46
Years post transplant
T-cell depletion
Positive for CD34mono
Negative
P=0.17
Years post transplant

Figure S2

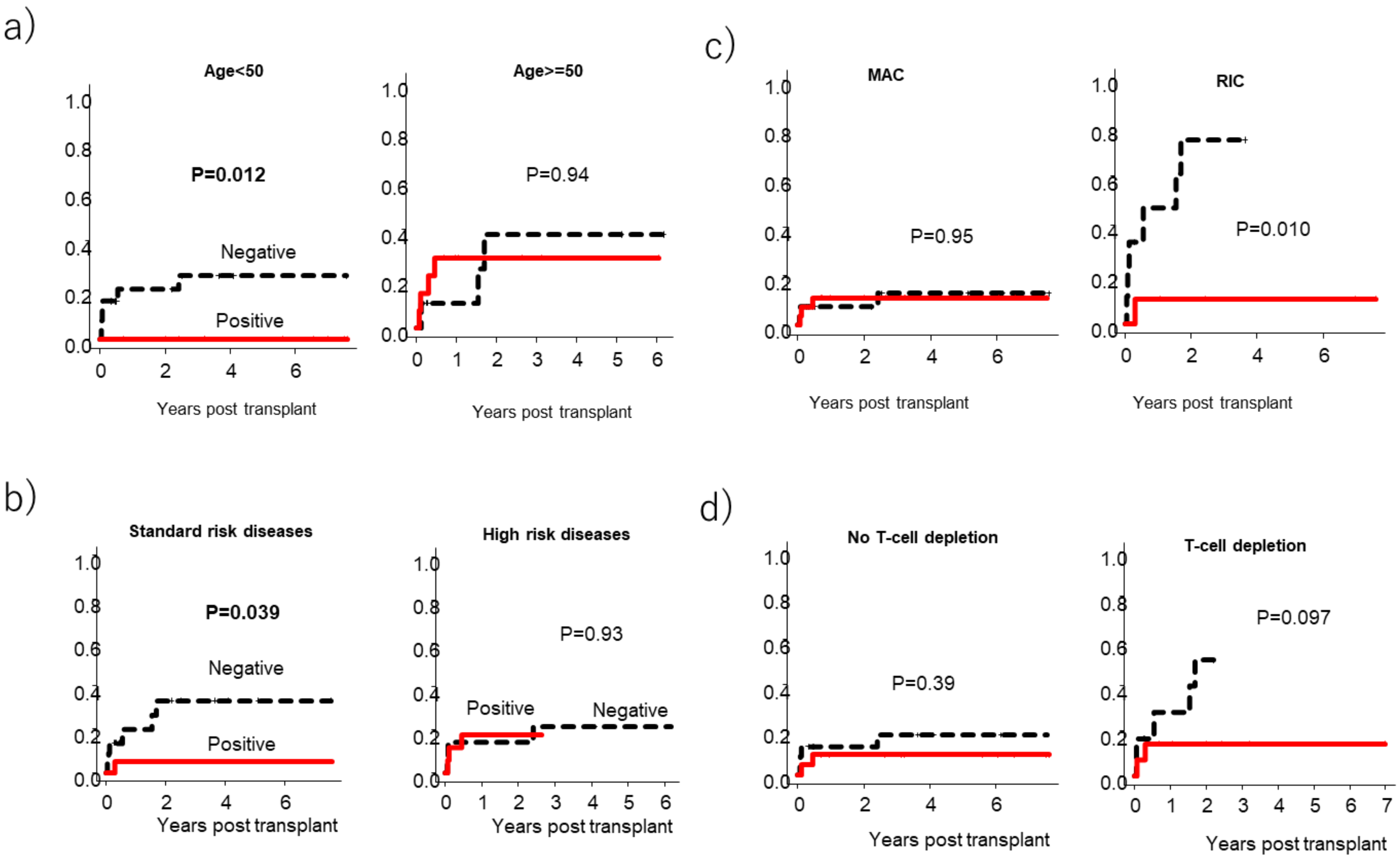
a)
Age<50
P=0.012
Negative
Positive
Years post transplant
Age>=50
P=0.94
Years post transplant
b)
Standard risk diseases
P=0.039
Negative
Positive
Years post transplant
High risk diseases
P=0.93
Positive
Negative
Years post transplant
c)
MAC
P=0.95
Years post transplant
RIC
P=0.010
Years post transplant
d)
No T-cell depletion
P=0.39
Years post transplant
T-cell depletion
P=0.097
Years post transplant

Figure S3

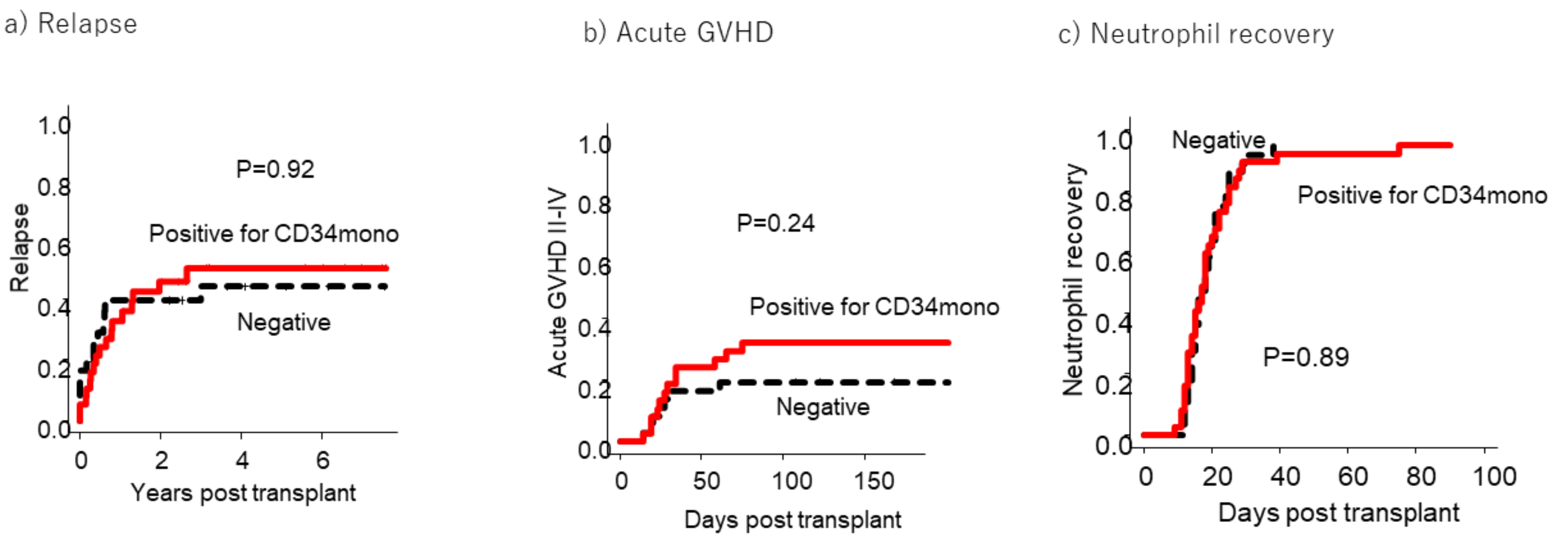
a) Relapse
P=0.92
Positive for CD34mono
Negative
Relapse
1.0
0.8
0.6
0.4
0.2
0.0
0
2
4
6
Years post transplant
b) Acute GVHD
P=0.24
Positive for CD34mono
Negative
Acute GVHD II-IV
1.0
0.8
0.6
0.4
0.2
0.0
0
50
100
150
Days post transplant
c) Neutrophil recovery
Negative
Positive for CD34mono
P=0.89
Neutrophil recovery
1.0
0.8
0.6
0.4
0.2
0.0
0
20
40
60
80
100
Days post transplant

Figure S4

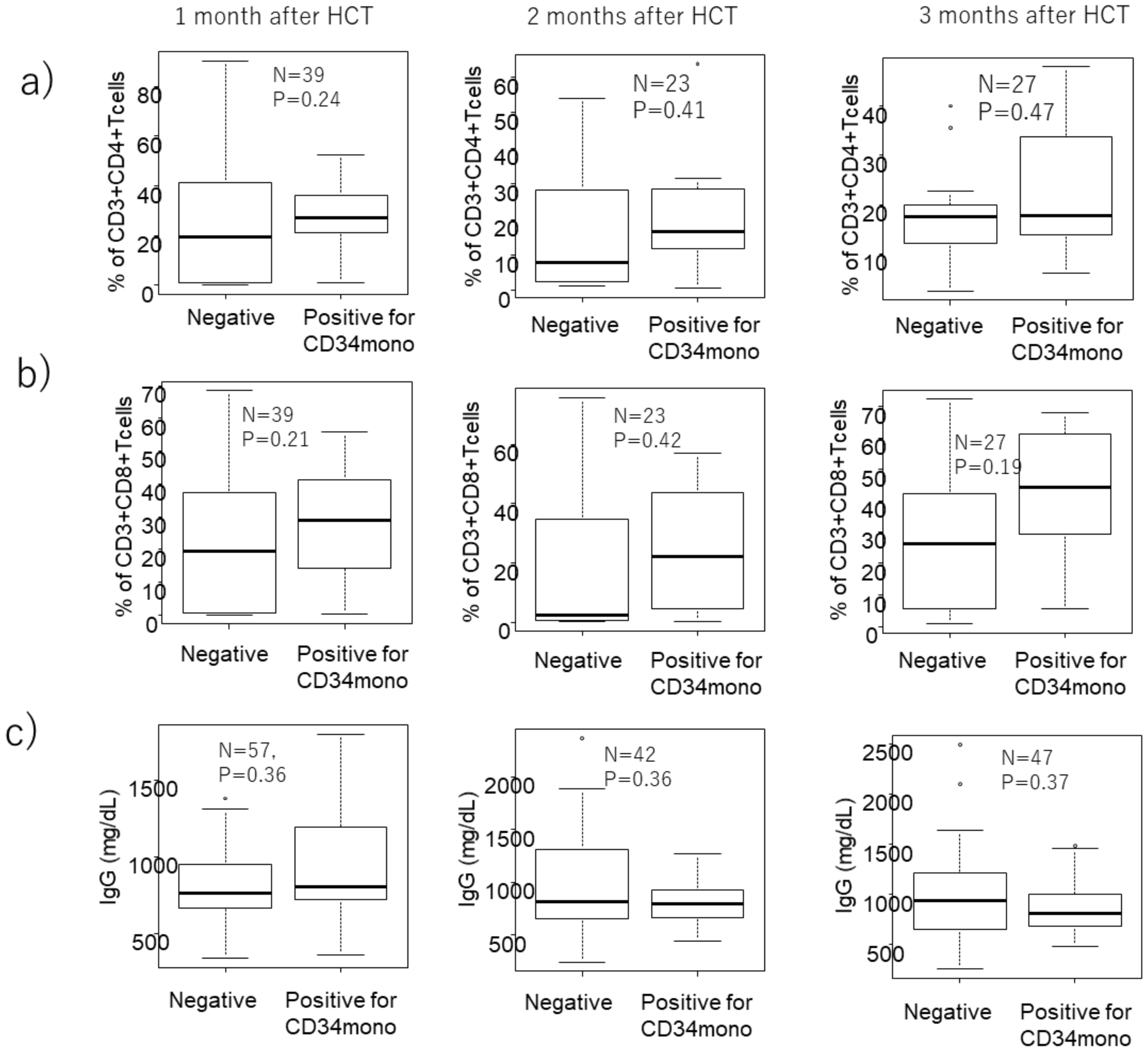
1 month after HCT
2 months after HCT
3 months after HCT
a)
b)
c)
% of CD3+CD4+Tcells
% of CD3+CD8+Tcells
IgG (mg/dL)
N=39
P=0.24
N=23
P=0.41
N=27
P=0.47
N=39
P=0.21
N=23
P=0.42
N=27
P=0.19
N=57,
P=0.36
N=42
P=0.36
N=47
P=0.37
Negative
Positive for CD34mono

There are several supplementary data files that cannot be embedded into the Word file.

Data file S1. Differentially expressed genes in CD34+mono compared with CD34+cells and con-mono.

Data file S2. GO biological process for differentially expressed genes in CD34+mono.

Data file S3. KEGG pathways for differentially expressed genes in CD34+mono.

Data file S4. A clinical dataset for survival analyses.
